# Promoting influenza prevention for elderly people in Hong Kong using health action process approach: study protocol

**DOI:** 10.1186/s12889-018-6146-6

**Published:** 2018-11-06

**Authors:** Chun-Qing Zhang, Ru Zhang, Pak-Kwong Chung, Yanping Duan, Joseph Tak fai Lau, Derwin King Chung Chan, Martin S. Hagger

**Affiliations:** 1 Department of Sport and Physical Education, Faculty of Social Sciences, Hong Kong Baptist University, Kowloon Tong, Kowloon, Hong Kong SAR, China; 2 Centre for Health Behaviours Research, JC School of Public Health and Primary Care, The Chinese University of Hong Kong, Hong Kong SAR, China; 3 School of Public Health, The University of Hong Kong, Hong Kong SAR, China; 4 Health Psychology and Behavioural Medicine Research Group, School of Psychology, Curtin University, Perth, Australia; 5 Faculty of Sport and Health Sciences, University of Jyväskylä, Jyväskylä, Finland

**Keywords:** Influenza prevention, Psychological theory, Behavior initiation, Behavior maintenance, Older adults

## Abstract

**Background:**

People 65 years or older are at greater risk of serious complications from the seasonal influenza compared with young. To promote elderly people’s behavioral compliance toward influenza prevention, the aim of the current project is to develop, implement, and evaluate a theory-based low-administration-cost intervention building on a leading psychological theory, the Health Action Process Approach (HAPA).

**Methods:**

The target group is Hong Kong Chinese elderly people aged 65 or older who rarely or never adopt any preventive actions. This project will be conducted in three phases over 24 months. In phase 1, intervention program will be developed building on the HAPA theoretical framework which comprises both the initiation and maintenance of influenza prevention behaviors. In phase 2, intervention will be implemented and evaluated using a randomized controlled trial, including: (a) behavior initiation only, (b) behavior initiation + behavior maintenance, and (c) control group. Both the initiation and maintenance components will comprise weekly-delivered telephone-based individual intervention sessions in 3 months. In phase 3, outcome evaluation of behavioral and psychological variables and process evaluation will be conducted. The effectiveness of the intervention will be analyzed using a series of linear mixed models on each behavioral and psychological outcome variable. Structural equation modelling will be used to test the hypothesized theoretical sequence in the HAPA model.

**Discussion:**

The proposed project is expected to design theory-based intervention materials to promote the influenza prevention behaviors in Hong Kong elderly people and provide information on its effectiveness and the potential changing mechanism of behavior initiation and maintenance.

**Trial registration:**

This randomized controlled trial was funded by the Health and Medical Research Fund (HMRF), Food and Health Bureau of the Government of the Hong Kong Special Administrative Region (Ref: 16151222) and was registered on 13/10/2017 at CCRB Clinical Trials Registry of the Chinese University of Hong Kong, a Partner Registry of a WHO Primary Registry (Ref: CUHK_CCRB00567).

## Background

Seasonal influenza can cause mild to severe illness, or even death. Each year, approximately 1 million deaths worldwide are estimated to be related to influenza [1]. According to the American Center for Disease Control and Prevention [2], certain groups of people, such as children under five, elderly people aged over 65, pregnant women, and people with certain chronic medical conditions, are at high risk of seasonal flu-related complications. It has been reported that Hong Kong elderly people (age above 65) are 14 times more likely than midlife adults (age between 40 and 65) to die from influenza [1]. Based on an assessment of weekly hospitalization counts in Hong Kong during 1996 to 2000, influenza was revealed to be significantly associated with hospitalization for acute respiratory disease, with large and noticeable rates of excess hospitalization for elderly people aged above 65 [3]. In peak influenza season, these individuals are more vulnerable to influenza. As such, elderly people are an ‘at risk’ population, given the fact that the population is, in general, aging and is going to increase massively in the future. This leads to the prevention of illness in this population a priority for governments, otherwise it’s going to cost health services a massive amount. To prevent infection with seasonal flu, people are advised to maintain some key prevention behaviors, such as wearing facemasks in enclosed public spaces, hand washing, using antibacterial sanitizers, or to consider having an influenza vaccination [4].

According to the concept of bounded rationality [5], individuals in influenza pandemic make decisions on health care and medication using heuristics, or rules based on past experiences and information instead of using rational decisions. Therefore, it is necessary to provide individuals a theory and evidence-based intervention to guide them form rational decisions when facing an influenza pandemic. Research adopting social psychological theories in influenza prevention has generally focused on influenza vaccination behaviors examining perceived severity, perceived barriers, perceived susceptibility, and perceived benefits, as well as social norms, intentions, worries and regrets towards vaccinations [6–11]. There are several theoretical models to explain the decisions and behaviors on influenza vaccination. For example, the health belief model [12] examines individuals’ perceptions and attitudes towards illnesses or health compromising behaviors in terms of three facets: individual perceptions (i.e., perceived susceptibility and perceived severity), modifying factors (i.e., perceived threat, environmental factors, and cues to action), and likelihood of action (i.e., perceived benefits and perceived barriers). Previous studies have provided initial support for the predictive value of these facets on individuals’ acceptance of influenza vaccination [6, 10]. Yet, the risk perception alone cannot fully explain preventive behaviors without considering the influence of outcome expectations and social norms, namely, social pressure of a normative status. The theory of planned behavior [13], on the other hand, explains the influences of individuals’ attitudes, subjective norms, and perceived behavioral control on the intentions and behaviors of influenza prevention. Intention is viewed as the most proximal determinant of individuals’ volitional behaviors. Research has preliminarily demonstrated significant associations between these beliefs and intentions to receive the influenza vaccine, which in turn predicted vaccination behaviors [9]; while non-intention to vaccination is significantly related to disbelief in the efficacy of, and negative attitudes toward vaccination as well as lack of perceived threat [7].

Although vaccination is an ideal way to prevent all types of influenza, numerous serotypes and the high variability of the influenza virus still challenge the uptake of influenza vaccines. In addition, everyday preventive actions, such as washing hands, avoid touching eyes, nose or mouth, and facemask wearing also play a very important role in prevention. Moreover, the relationship between vaccination and other influenza preventive behaviors (e.g., personal hygiene practices) is still unknown. Regarding as one of the main factors contributing to the transmission of influenza within the community, low behavioral compliance to these recommended health actions are frequently reported [14] and this might be explained as acceptance of vaccination accidently warrant people a false information/belief of free of being infected, which further causing them to lower their behavioral compliance. Nonetheless, researchers have begun to investigate the psychological factors associated with the lack of behavioral compliance toward influenza prevention [6, 15]. For example, consistent with the health belief model, the reason people fail to, or refrain from, wearing facemasks might be due to a lack of perceived susceptibility, cues to action, and perceived benefits, rather than lack of perceived severity and perceived barriers [15].

Although both the health belief model and theory of planned behavior could somewhat explain the complex cognitive interactions in influenza prevention, the factors of post-intentional volitional stage have been inadequately addressed which play an important role in the maintenance of preventive behaviors. Although the motivational paths to intentions have been well-established with large effect sizes, the prediction from intention to health behaviors always fail, a phenomenon called “the intention-behavior gap” [16]. Based on the model of action phases [17] and social-cognitive theory [18], the health action process approach (HAPA) [16] was developed by integrating these two theoretical approaches. The HAPA stipulates a two-phase approach to action, a motivational phase and an implemental or ‘planning’ phase. Motivation is considered a necessary but not sufficient condition for action initiation and persistence; people also need to identify effective plans to enact their intentions or motives. Interventions should, therefore, target both motivational and implemental phases [19]. In other words, it suggests a distinction between (a) pre-intentional motivational processes that lead to a behavioral intention, and (b) post-intentional process that lead to actual health-related behavior. The HAPA is, therefore, an appropriate approach to explain the processes that lead to motivated behavior and its enactment in health contexts, including the influenza vaccination behaviors [20]. It has also been demonstrated applicable in the local context of Hong Kong for other health-related behaviors such as the condom use for men who have sex with men [21].

The current project therefore will develop a HAPA-based intervention to promote influenza prevention behaviors in Hong Kong older adults. In order to promote the initiation and maintenance of influenza prevention behaviors, it will comprise intervention components targeting both motivation and planning. In the behavior initiation stage, three key psychological variables have been associated with increased motivation toward influenza prevention: risk perception, action self-efficacy, and outcome expectancy. The intervention components that have been shown to target and effect change in each of these variables are information provision (risk perception, action self-efficacy, and positive outcome expectancy), goal setting (action self-efficacy), and mental simulations (positive outcome expectancy). In the behavior maintenance stage, different strategies will be used which include the maintenance/relapse prevention self-efficacy, coping planning, and maintaining the satisfaction and enjoyment of the behavior change. The components targeting increased planning are action plans or implementation intentions [19]. Comparing to the behavior initiation stage which focuses on approaching a more favorable health stage, the maintenance stage help participants avoid reverting to a less favorable non-intending status [22].

### Amis of the current study

In order to increase influenza prevention behaviors in Hong Kong older adults (aged 65 years and over), the aim of this project is to develop, implement, and evaluate a HAPA-based intervention, which comprises two main components of a health behavior change, namely, behavior initiation and behavior maintenance. Four specific objectives will be tested: (a) design and develop an intervention to promote influenza prevention behaviors consistent with guidelines of Department of Health in non-intending older adults; (b) imply the intervention in a sample of older adults in Hong Kong who reported never or rarely adopt influenza preventive measures during the past year using a randomized controlled trial; (c) evaluate the effectiveness and a 6-month sustainability of the intervention to change primary outcome variables (i.e., washing hands, avoid touching eyes, nose or mouth, facemask wearing, and vaccination); and (d) identify the psychological variables (i.e., intention, action and coping planning, maintenance and recovery self-efficacy) responsible for explaining the effect of the intervention.

Five hypotheses will be tested in this project: (a) participants in the two intervention groups (i.e., behavior initiation + behavior maintenance; behavior initiation only) will have significantly higher rates of influenza prevention behaviors relative to participants allocated to the control group; (b) participants in the “behavior initiation + behavior maintenance” intervention group will have significantly higher participation in influenza prevention behaviors relative to participants allocated to the “behavior initiation only” intervention group; (c) participants in the two intervention groups (i.e., behavior initiation + behavior maintenance; behavior initiation only) will report significantly greater action self-efficacy, outcome expectancy, risk perception, and intention than participants in the control group. Participants in the “behavior initiation + behavior maintenance” intervention group will report stronger action and coping planning, maintenance and recovery self-efficacy, and social support than participants in the “behavior initiation only” intervention group and the control group; (d) participants in the “behavior initiation + behavior maintenance” intervention group will report significantly greater action self-efficacy, outcome expectancy, risk perception, and intention, and stronger action and coping planning, maintenance and recovery self-efficacy, and social support than participants in the “behavior initiation only” intervention group; (e) consistent with the propositions of the health action process approach, intention, action and coping planning, maintenance and recovery self-efficacy will mediate the effect of the intervention condition on influenza prevention behaviors.

## Methods

### Participants

Participants in the current study will comprise Hong Kong Chinese older adults. Participants will be eligible if they are 65 years or over; retired or homemakers; willing to be randomly assigned to experimental or control groups; able to understand the study rationale; a first-language Cantonese-speaker; and, most importantly, non-vaccinated within 1 year and report never or rarely adopting any of the influenza prevention behaviors listed by the research group. Participants who report more frequent preventive behaviors will not be invited to attend the preliminary session. We will also exclude older adults who are dementia as well as who are fragile to move.

### Design

**Fig. 1:**
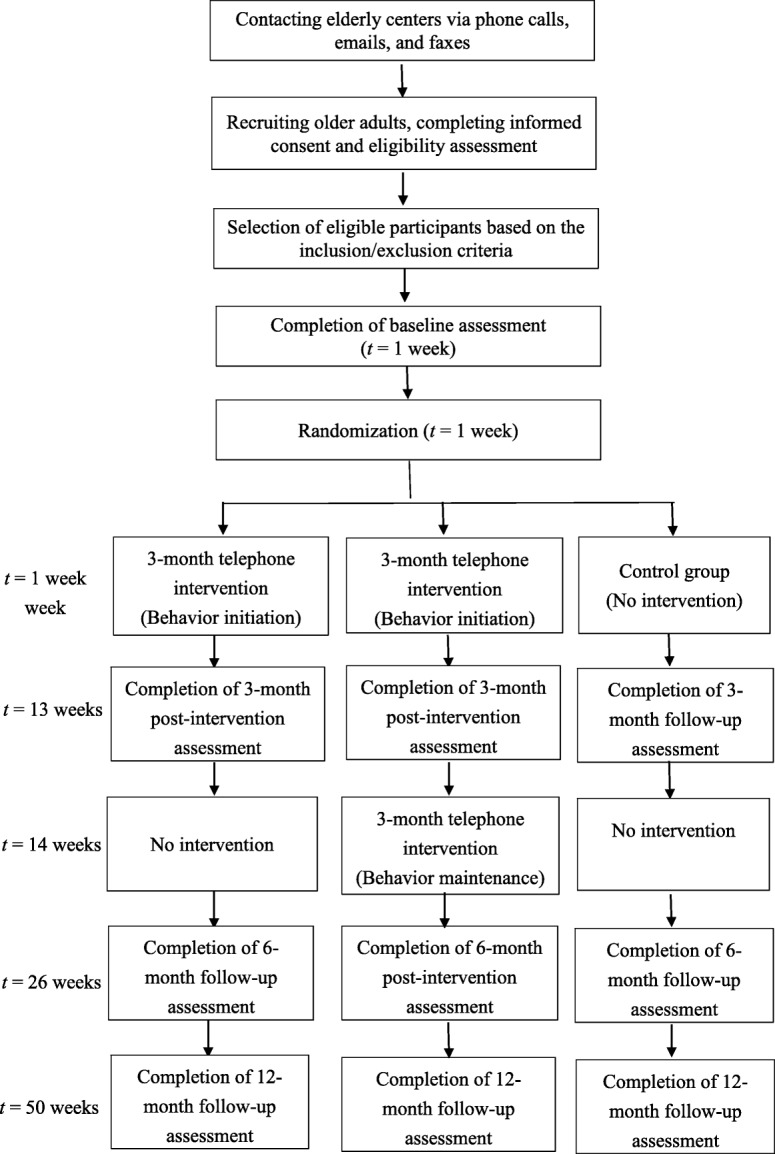
Study design

This project will be conducted in three phases over 24 months in a sample of older adults from Hong Kong. Phase 1 (Months 1 to 4) – Development of intervention using the HAPA theory and preparation for data collection including participant recruitment and preparation of materials and measures. Recruitment will overlap with Phase 2 until the required sample size has been recruited. Phase 2 (Months 4 to 20) – Implementation of the intervention including baseline measures, intervention administration through telephone, ongoing follow-up outcome (See Fig. 1). Phase 3 (Months 21 to 24) – Evaluation of intervention through: (a) evaluating the immediate impact through effects on outcomes at 6-month post-intervention; (b) evaluating the long-term impact of the intervention through effects on outcomes at 12-month follow up; and (c) assessing the compliance of the participants with the intervention.

### Phase 1: intervention development

The rationale of the intervention development will be in developing increased motivation to adopt influenza preventive behaviors in Hong Kong older adults and assisting them in converting their motives into action and maintaining the behavior [16, 22]. According to the HAPA theory, motivation is a necessary but not sufficient condition for action initiation and persistence; people also need to identify effective plans to enact their intentions or motives. The protocol will therefore include intervention components that promote motivation (action self-efficacy, risk perception, and outcome expectancy) and planning (action and coping planning, maintenance and recovery self-efficacy).

Aims of the first phase are: (i) develop the content of the HAPA-based intervention to promote the influenza prevention behaviors of Hong Kong older adults building on our previous projects on influenza and influenza prevention; (ii) design an intervention training manual so that the intervention can be administered consistently across groups and replicated by others; (iii) develop the intervention delivery protocol consistent with the design of randomized controlled trial; (iv) develop measures of key outcome variables to be used to evaluate the effectiveness of the intervention; and (v) confirm participation of elderly centers (i.e., government-established elderly care facilities) to be involved in the intervention and secure ethical clearance for the study. Prior to the start of the project upon funded, the study protocol has been registered in CCRB Clinical Trials Registry of the Chinese University of Hong Kong, a Partner Registry of a WHO Primary Registry (Ref: CUHK_CCRB00567). Main components and example content of the study protocol are listed below.

#### Behavior initiation

Several motivational strategies of behavior initiation will be included: (a) The information-giving component will include a slide presentation from the facilitator to the group promoting the advantages, and negating the disadvantages, of influenza prevention for health and outlining the risk levels of influenza for older adults (targeting risk perception); a ‘how to’ education unit aimed at providing information about how to conduct influenza prevention behaviors in certain scenario, such as covering both mouth and nose when wearing facemasks instead of solely on month in a crowded place during peak of flu (targeting action self-efficacy); and a brief example of an influenza prevention program and possible outcomes and gains of engaging in regular influenza prevention behaviors (targeting positive outcome expectancy); (b) In the goal-setting exercise, participants will be given guidance on how to set a goal of adopting influenza prevention (targeting action self-efficacy). They will be told that the aim is to develop personal goals for increasing their influenza prevention behaviors, and participants will be encouraged to set goals realistic short- and long-term goals; (c) In the ‘mental simulation’ activity, participants will be asked to visualize the actions they would need to perform to achieve their goals and how they would feel once they had achieved those goals (targeting positive outcome expectancy); and (d) In the implementation intention activity participants will be asked to set specific action plans of when, where, and in which situations they will adopt influenza prevention behaviors and tying it in with everyday occurrences to remind them to implement their plan (targeting planning).

#### Behavior maintenance

Several strategies will be introduced in the maintenance session and be compared with the concepts at the initiation session [22]: (a) maintenance/relapse prevention self-efficacy instead of action self-efficacy, that is, building the confidence in overcoming barriers to continue the influenza prevention behaviors (e.g., avoiding embarrassment of wearing a facemask in a public place); (b) coping planning should be emphasized rather than simply focused on action planning, this is because that the established new behavior patterns might be threatened by previous habits and some specific situations; (c) the feelings of enjoyment/satisfaction in complying to the influenza prevention behaviors will be emphasized rather than the expectations of the benefits of influenza prevention behaviors, this because perceived satisfaction has long been demonstrated as a significant predictor of behavior maintenance; and (d) participants will be encouraged to actively seeking social support from their networks, which will replace the support from the project facilitator when the project is withdrawn.

#### Control group

Participants randomly assigned to the control group will not receive the behavior-change components of the telephone-delivered intervention sessions. Indeed, participants in the control group will receive some general information on influenza prevention in the group-delivered preliminary session. But, the behavior-change aspects of the information-giving component, goal-setting, mental-simulation, and implementation intention components will be absent from the control group as they will not receive the telephone-delivered intervention sessions. The part-time research assistants will be provided with training and a feedback session to ensure consistency in delivering the intervention.

### Phase 2 – implementation of the intervention

#### Sample size

We will assume a small effect size (Cohen’s *d* = .20) for the effect of the intervention in order to calculate a conservative post-intervention sample size sufficient to detect an effect. G*Power analysis using the between-factor (i.e., 3 groups) repeated measures (i.e., 4 phases; baseline, 3-month, 6-month, and 12-month measurements) multivariate analysis of variance (MANOVA) indicates that a sample size of 65 in each group is required at follow-up in order to detect a small effect size (*d* = .20) in the outcome variables with power (beta) set at .90 and significance level (alpha) set at .017 (i.e., .05/3). Based on conservative estimate of 25% attrition rates, we aim to recruit 261 participants at baseline (*n* = 87 participants per group).

#### Recruitment strategy

Given the links the research team has established with the elderly centers (including District Elderly Community Centers, Neighborhood Elderly Centre) in our previous projects, we will make contacts (i.e., phones, emails, and faxes) with center-in-charges and employ their support in championing recruitment to the study. We plan to recruit participants who are registered as members of the elderly centers at the three main territories of Hong Kong (i.e., Hong Kong Island, Kowloon, and New Territories).

#### Preliminary group-based education session

Interested participants will be invited to attend an initial session where they will be screened for eligibility and be provided with full details of the study and an information and consent form pack. Participants will also be invited to participate in a one-off group education session on the general information about types of influenza, peak influenza season, routes of influenza transmission, and influenza preventive behaviors. Participants will then be randomized into one of the three conditions: (a) “behavior initiation” and (b) “behavior initiation + behavior maintenance”, and (c) “control group”. Randomisation will be conducted by a research staff who is blind to the composition of the participants using the online randomisation tool. Participants will be blinded to the intervention allocation, while the part-time research assistants will be blinded to the research purpose.

#### Baseline measures

Participants will be asked to complete baseline demographic and psychological measures with the assistance of student helpers. Participants will be required to complete a questionnaire comprising: (a) demographic and economic variables (age, gender, education level, marital status and living arrangement, household income); (b) psychological variables using validated self-report measures of risk perception, outcome expectancies, action and coping self-efficacies, action and coping planning, and intention, with Chinese versions of these measures have been validated and used in a previous study [23], while items of recovery and maintenance self-efficacy [24] will be translated and back-translated and validated into Chinese prior to the start of study; and (c) influenza prevention behavioral variables (i.e., washing hands, avoid touching eyes, nose or mouth, and facemask wearing) will also be provided. In terms of the hand washing behavior, two situation-specific questions will be asked including (a) return to home and/or elderly center after being out and (b) before touching food. In terms of the behaviors of avoid touching eyes, nose or mouth, we ask how many times that participants be aware of and successfully inhibiting themselves touching their eyes, nose or mouth before they have washed their hands, particularly after they have been touching other objects that might lead them to exposure risk (e.g., stair rails and bannisters, door handles, chairs and tables, safety rails on the MTR etc.). In terms of the facemask wearing behaviors, we ask participants their facemask wearing behaviors when (a) in direct contact with people, (b) in crowded places such as shopping malls and the MTR. Participants will be informed that they will receive telephone calls in three randomly-selected days in the following 9 days asking them to report whether they have conducted the three influenza prevention behaviors. Participants’ self-reported clinical influenza episodes as well as flu-like symptoms during the past 3 months will also be collected.

#### Telephone-delivered individual behavior initiation sessions

Participants in (a) “behavior initiation” and (b) “behavior initiation + behavior maintenance” intervention groups will receive telephone-delivered intervention sessions at weekly intervals throughout the course of behavior initiation for 3 months. The purpose of these is to serve to remind participants of their influenza prevention goal and their action plans to implement them, mainly focused on the motivational stage. The intervention sessions will be administered by telephone lasting 10 to 20 min, and will be audio-recorded with the permission from participants for fidelity checks. To the extent that participants will have talked about their appropriate influenza prevention goals in the goal setting, mental simulation, and action planning exercises, step-by-step personal instructions and feelings about pursuing their goal, and when and where they will pursue their goals. Participants in the control group will not receive the telephone-delivered intervention sessions. Part-time research assistants will be recruited to deliver the telephone intervention sessions appropriately 10–15 min. They were be trained by, and receive feedback from, the PI and project coordinator and practice with each other in simulated scenarios.

#### Telephone-delivered individual behavior maintenance sessions

Following the behavior initiation session, all participants will be asked to report the three influenza prevention behaviors via telephone calls in three randomly-selected days in 9 days, clinical influenza episodes as well as flu-like symptoms in the past 3 months, and then they will again be invited to complete the same set of psychological outcome measures administered at baseline. Thereafter that only participants allocated to the “behavior initiation + behavior maintenance” intervention group will continue to receive telephone-delivered intervention sessions at weekly intervals for 3 months. The sessions will focus on strategies of behavior maintenance at the volitional stage.

#### Follow-up measures

At the 3-month (i.e., the middle time point of the intervention after the behavior initiation session), 6-month (post-intervention), and 12-month follow-up occasions, participants will receive the same set of psychological outcome measures administered at baseline. They will also be asked to report the three influenza prevention behaviors (i.e., washing hands, avoid touching eyes, nose or mouth, and facemask wearing) as well as whether they get vaccinated via telephone calls in three randomly-selected days in 9 days. Participants’ self-reported clinical influenza episodes as well as flu-like symptoms during the past 3 months will also be collected. Participants will be reminded of their follow-up visit by telephone in advance of their visit. In order to minimize attrition, in particular participants from the control group, a fixed amount of monetary reward will be provided to those who completed all assessments.

### Phase 3 – evaluation of the intervention

#### Process evaluation

We will include brief self-report measures at data collection sessions to gauge participants’ views of the acceptability of the intervention components and the intervention itself as well as measures of behavioral and psychological variables. This will provide useful data on the level of intrusion experienced by the participants and an indication of its acceptability. Questionnaire measures of planning will also provide evidence of compliance. In addition, we will conduct a content analysis of the scripts of the audio files recorded during the telephone-delivered intervention sessions, in order to confirm their compliance with the intervention. This will permit the formal evaluation of intervention fidelity. As part of the fidelity checks and to minimize the potential influence of contamination and spill-over, we will ask participants whether they are aware of any eligible participants from the other intervention or control groups are their friends they interacted with, whether they have enrolled in some influenza prevention classes offered by their centers, as well as whether they are aware of any special effort has been provided by the center to promote influenza prevention in the period of intervention. In addition, we will encourage participants not to share their experiences with other potential participants. Moreover, we will track the impact of the influence of the potential new pandemic if any by asking participants their awareness and feelings.

#### Outcome evaluation

Psychological data will be collected at an isolated room provided by the elderly center during the baseline, the 3-month (the middle time point of intervention), the 6-month (post-intervention), and the 12-month follow-up occasions, whereas the three influenza prevention behaviors variables (i.e., washing hands, avoid touching eyes, nose or mouth, and facemask wearing) and vaccination will be collected via telephone calls in three randomly-selected days in nine. Participants’ self-reported clinical influenza episodes as well as flu-like symptoms during the past 3 months will also be collected.

#### Data analysis

Data will be stored in password-protected spreadsheets saved on a computer of a member of the research team. Data will be initially treated for missing values with multiple imputation. The distribution of the data will be examined that the skewed data will be log-transformed and replaced with median (interquartile range). All analyses will be conducted using full intention-to-treat analyses with scores on dependent variables for non-compliers carried over using the last-observation carried forward method. Independent samples t-tests and chi-square analysis will be used to test whether there are significant differences between non-compliers and compliers. We will analyze the effectiveness of the intervention using a series of linear mixed models on each behavioral and psychological outcome variable. In the analyses, four phases will be the within-participants factor and three intervention conditions will be the between-participants factor. Baseline scores for the dependent variables will be included as covariates in the analysis with salient demographic variables: age, gender, marital status, education level, and socioeconomic status. We will use Akaike and Bayesian information criteria to assess whether a compound symmetry covariance structure will provide the best model fit of each outcome variable. The phase (i.e., time of measurement) by intervention condition (i.e., behavior initiation + behavior maintenance; behavior initiation only; and control group) interaction will be the central test. Beyond main effects of the intervention, we will also test whether there are any statistically significant interactions between the intervention condition and the time factor. For all tests, the Cohen’s d effect size will be calculated associated with a 95% confidence interval. Structural equation modelling (SEM) will be used to test the hypothesized theoretical sequence in the HAPA model [25]. In the analyses, the effect of the intervention group conditions will be included using a dummy coded variable along with the demographic and baseline variables. The 95% bias corrected (BC) bootstrapping method (with *n* = 5000 bootstrap resamples) was subsequently employed to test the indirect effects of mediators (e.g., intention, maintenance and recovery self-efficacy, action and coping planning) (25). The CONSORT Extension for Nonpharmacological Treatment Interventions will be followed to report the results.

## Discussion

As a subtropical city, Hong Kong is located at the hypothetical epicenter of influenza pandemics [1, 3]. Despite the widespread influenza prevention measures suggested by various national and regional centers for disease control and prevention, relatively little research has been done to promote the adoption of preventive behaviors based on the psychosocial theories, as compared to the basic research for vaccination development and large-scale telephone interviews [14, 15]. Although vaccination is the most effective way of preventing influenza and much existing research focuses on the influence of psychosocial variables for vaccination rather than daily protective measures, effective daily preventive measures are also important [4]. Most importantly, even the vaccination is not able to guarantee full protection from influenza. Therefore, the current 24-month project aimed to develop a theory-based intervention materials for influenza prevention behaviors and examine its effectiveness in a sample of Hong Kong elderly people. Particular emphasis will be on the emphasis on both the initiation and maintenance of influenza prevention behaviors [17].

The key benefit of the current research will be development and application of theory-based intervention materials to promote influenza prevention of Hong Kong older adults. The materials developed for the current project will supplement and extend existing educational materials such as those available from the Centre for Health Protection of Hong Kong, which largely focus on the provision of information rather than how to help older adults to initiate and maintain the influenza prevention behaviors. The impact of the project findings will be maximized by dissemination to key academic, policy, and practitioner groups.

## Abbreviations

BC: Bootstrapping
CCRB: Centre for Clinical Research and Biostatistics
CONSORT: Consolidated Standards of Reporting Trials
HAPA: Health action process approach
MANOVA: Multivariate analysis of variance
MTR: Mass Transit Railway
SEM: Structural equation modelling
WHO: World Health Organization

## References

[1] Wong CM, Chan KP, Hedley AJ, Peiris JSM (2004). Influenza-associated mortality in Hong Kong. Clin Infect Dis.

[2] Centers for Disease Control and Prevention. Morbidity and Mortality Weekly Report (MMWR): Estimates of deaths associated with seasonal influenza - United States, 1976–2007. https://www.cdc.gov/mmwr/pdf/wk/mm5933.pdf. Accessed 27 Aug 2010. 20798667

[3] Wong CM, Yang L, Chan KP, Leung GM, Chan KH, Guan Y (2006). Influenza-associated hospitalization in a subtropical city. PLoS Med.

[4] Agüero F, Adell MN, Giménez AP, Medina MJL, Continente XG (2011). Adoption of preventive measures during and after the 2009 influenza a (H1N1) virus pandemic peak in Spain. Prev Med.

[5] Kahneman D (2003). A perspective on judgment and choice: mapping bounded rationality. Am Psychol.

[6] Nexøe J, Kragstrup J, Søgaard J (1999). Decision on influenza vaccination among the elderly a questionnaire study based on the health belief. Scand J Prim Health Care.

[7] Byrne C, Walsh J, Kola S, Sarma KM (2012). Predicting intention to uptake H1N1 influenza vaccine in a university sample. Br J Health Psychol.

[8] Liao Q, Cowling BJ, Lam WWT, Fielding R (2011). The influence of social-cognitive factors on personal hygiene practices to protect against influenzas: using modelling to compare avian a/H5N1 and 2009 pandemic a/H1N1 influenzas in Hong Kong. Int J Behav Med.

[9] Liao Q, Cowling BJ, Lam WWT, Fielding R (2011). Factors affecting intention to receive and self-reported receipt of 2009 pandemic (H1N1) vaccine in Hong Kong: a longitudinal study. PLoS One.

[10] Liao Q, Wong WS, Fielding R (2013). How do anticipated worry and regret predict seasonal influenza vaccination uptake among Chinese adults?. Vaccine.

[11] Prati G, Pietrantoni L, Zani B (2011). A social-cognitive model of pandemic influenza H1N1 risk perception and recommended behaviors in Italy. Risk Anal.

[12] Janz NK, Becker MH (1984). The health belief model: a decade later. Health Educ Q.

[13] Ajzen I (1991). The theory of planned behavior. Organ Behav Hum Decis Process.

[14] Lau JTF, Griffiths S, Choi KC, Lin C (2010). Prevalence of preventive behaviors and associated factors during early phase of the H1N1 influenza epidemic. Am J Infect Control.

[15] Tang CS, Wong C (2004). Factors influencing the wearing of facemasks to prevent the severe acute respiratory syndrome among adult Chinese in Hong Kong. Prev Med.

[16] Schwarzer R (2008). Modeling health behavior change: how to predict and modify the adoption and maintenance of health behaviors. Appl Psychol.

[17] Heckhausen H, Gollwitzer PM (1987). Thought contents and cognitive functioning in motivational versus volitional states of mind. Motiv Emot.

[18] Bandura A (1977). Self-efficacy: toward a unifying theory of behavioral change. Psychol Rev.

[19] Hagger MS, Luszczynska A (2014). Implementation intention and action planning interventions in health contexts: state of the research and proposals for the way forward. Appl Psychol.

[20] Ernsting A, Gellert P, Schneider M, Lippke S (2013). A mediator model to predict workplace influenza vaccination behaviour–an application of the health action process approach. Psychol Health.

[21] Teng Y, Mak WW (2011). The role of planning and self-efficacy in condom use among men who have sex with men: an application of the health action process approach model. Health Psychol.

[22] Voils CI, Gierisch JM, Yancy WS, Sandelowski M, Smith R, Bolton J, Strauss JL (2014). Differentiating behavior initiation and maintenance: theoretical framework and proof of concept. Health Educ Behav.

[23] Duan Y, Lippke S, Wagner P, Brehm W (2011). Testing two stage assessments in a Chinese college student sample: correspondences and discontinuity patterns across stages. Psychol Sport Exerc.

[24] Luszczynska A, Schwarzer R, Lippke S, Mazurkiewicz M (2011). Self-efficacy as a moderator of the planning–behaviour relationship in interventions designed to promote physical activity. Psychol Health.

[25] Lau RS, Cheung GW (2012). Estimating and comparing specific mediation effects in complex latent variable models. Organ Res Methods.

